# Effects of Exposure to Micro- and Nanoplastics on Endometrial Injury and Adverse Pregnancy Outcomes: Evidence Integration Using the Targeted Risk Assessment of Environmental Chemicals Framework

**DOI:** 10.3390/toxics14080677

**Published:** 2026-07-31

**Authors:** Haofei Shen, Nan Jiang, Ahui Liu, Na Wang, Jiawei Gao, Xiaoyan Sun, Xiaoling Ma, Xiaorong Luo

**Affiliations:** 1 Reproductive Medicine Center, The First Hospital of Lanzhou University, Lanzhou 730000, China; 715106922@163.com (H.S.); maxl2005@163.com (X.M.); 2 The First Clinical Medical College, Lanzhou University, Lanzhou 730000, China; 15542356272@163.com (N.J.); wangna2021@lzu.edu.cn (N.W.); gjw2233123456@163.com (J.G.); 3 Reproductive Medicine Center, Xi’an People’s Hospital (Xi’an Fourth Hospital), Xi’an 710000, China; liuah1218@163.com; 4 The Affiliated Suzhou Hospital of Nanjing Medical University, Suzhou Municipal Hospital, Gusu School, Nanjing Medical University, Suzhou 215000, China

**Keywords:** MNPs, endometrial receptivity, adverse pregnancy outcomes, risk assessment, TRAEC strategy

## Abstract

Micro- and nanoplastics (MNPs) are emerging environmental pollutants, but evidence concerning their effects on endometrial injury and adverse pregnancy outcomes remains limited. Therefore, we employed the Targeted Risk Assessment of Environmental Chemicals (TRAEC) strategy to integrate evidence concerning endometrial injury and adverse pregnancy outcomes associated with exposure to MNPs. Following targeted screening, 21 unique studies yielded 24 literature-derived evidence entries. Together with one additional in vitro evidence item from the present study, 25 evidence items were included in the final CES calculation. The available evidence was quantitatively integrated across four predefined TRAEC dimensions: Reliability Scores, Weight of Concentrations, Risk Intensity, and Correlation Scores. Under the tested in vitro conditions, exposure to PS-NPs increased intracellular ROS accumulation, reduced mitochondrial membrane potential, promoted apoptosis, and decreased the expression of key endometrial receptivity proteins, namely HOXA10, FOXO1, and ITGB3, in primary human endometrial stromal cells. Based on this combined evidence pool, the final Comprehensive Evidence Score was 7.67, corresponding to a preliminary moderate-risk classification within the predefined TRAEC framework. In summary, the TRAEC strategy provides a structured approach for integrating multi-level evidence and deriving a framework-specific health-risk classification for exposure to MNPs.

## 1. Introduction

Since the 1950s, the global plastics industry has experienced explosive growth. By 2017, global plastic production had reached 348 million tons, and it is projected to surge to 33 billion tons by 2050 [1]. Plastics are widely used in various fields such as packaging, textiles, construction, healthcare, and consumer goods due to their lightweight, durable, and low-cost properties [2]. However, this large-scale production and use, coupled with their extremely difficult natural degradation characteristics, has led to serious global plastic pollution problems. Waste plastics enter the ocean, soil, fresh water, and even the air through a variety of ways and are decomposed into smaller particles under physical, chemical, and biological effects, namely microplastics (MPs, 100 nm–5 mm in diameter) and nanoplastics (NPs, <100 nm in diameter) [3]. Human exposure to micro- and nanoplastics (MNPs) may occur through diet, inhalation, and other routes [4,5]. MNPs have been detected in several human tissues and biological samples, including blood, lung, liver, kidney, and brain samples [6,7,8]. MPs have also been reported in human female reproductive tissues, while experimental studies suggest that selected particles may cross biological barriers and affect reproductive tissues [9]. Experimental evidence further suggests that exposure to MNPs may alter endometrial function and implantation-related processes through inflammatory and endocrine pathways [10]; however, causal effects on human reproductive outcomes have not been established.

The endometrium plays a central role in women’s reproductive health and pregnancy. In each menstrual cycle, the endometrium will undergo changes such as proliferation, secretion, and shedding under the action of estrogen and progesterone, preparing for possible embryo implantation. The key to this process lies in the ‘receptivity’ of the endometrium; that is, the mature endometrium during the implantation window allows embryonic trophoblast cells to attach to the endometrial epithelium and further invade the stroma and blood vessels [10]. Once the embryo is successfully implanted, the endometrium will undergo further decidualization, providing nutrition, oxygen, and immune protection for early embryos [11]. The normal endometrial function is closely related to a variety of adverse pregnancy outcomes. When endometrial receptivity is impaired, the best-quality embryos are also difficult to implant, which is one of the main causes of in vitro fertilization-embryo transfer (IVF-ET) failure and unexplained infertility [12]. Endometrial dysfunction is also closely related to a variety of common gynecological diseases, such as endometriosis, adenomyosis, and endometrial polyps [13,14]. Studies have shown that pregnant women are highly sensitive to environmental pollutants. At this time, pollutants such as MNPs are more likely to cross the placental barrier into the fetus and amniotic fluid, may induce oxidative stress and mitochondrial dysfunction and may be associated with adverse pregnancy outcomes [9].

With advances in analytical technologies, several studies have detected and characterized MPs in human endometrial tissue using Raman spectroscopy, laser direct infrared spectroscopy (LDIR), and pyrolysis–gas chromatography/mass spectrometry (Py-GC/MS) [15,16]. One study detected MPs composed mainly of polyamide (PA), polyurethane (PU), and polyethylene terephthalate (PET), with particle sizes ranging from 2 μm to 200 μm, in the endometrial tissues from 22 women [17]. Another study performed the first quantitative analysis of microplastics in the endometrium, finding a median abundance of 21 particles per 100 mg of tissue, with 88.35% of particles measuring between 20 and 100 micrometers. This study also identified certain lifestyle habits (such as specific drinking water habits and chewing gum) as significantly associated with higher microplastic exposure levels [15]. In a three-dimensional human endometrial model, exposure to PS-MPs reduced epithelial barrier function, activated TGF-β/SMAD signaling, and increased the deposition of type I, III, and IV collagen, indicating fibrotic remodeling under the tested conditions [18]. Another study reported higher MP abundance in endometrial polyps than in normal endometrium. In vitro, PS microspheres promoted the proliferation, migration, and invasion of endometrial stromal cells via PI3K/AKT activation, suggesting a potential role in polyp development [16]. However, the study did not establish whether MPs were encapsulated, and passive accumulation in pre-existing polyps cannot be excluded; thus, the direction of causality remains uncertain.

Established human health risk-assessment frameworks, such as those developed by the World Health Organization (WHO) and the US Environmental Protection Agency (US EPA), typically address hazard identification, dose–response assessment, exposure assessment, and risk characterization [19,20]. OECD Integrated Approaches to Testing and Assessment (IATA) combine multiple sources of information to address a defined hazard, safety-assessment, or regulatory question [21]. However, for emerging contaminants such as MNPs, quantitative risk characterization remains constrained by the heterogeneity of particle size, polymer composition, morphology, and surface properties, together with limited quantitative information linking external human exposure to internal tissue doses and adverse outcomes [9]. TRAEC was introduced through initial application studies in 2024 and subsequently refined as a standardized, health outcome-oriented framework for integrating epidemiological, in vivo, and in vitro evidence [22,23]. It quantitatively integrates study reliability, concentration-related information, effect intensity, and association direction to generate a framework-specific risk classification. In an initial PFAS neurodevelopment case study, TRAEC classifications showed concordance with evaluations using ToxRTool, SciRAP, the OHAT risk-of-bias tool, and IRIS-based approaches [22]. However, this comparison provides preliminary case-based methodological support rather than comprehensive external validation. Accordingly, TRAEC is applied here as a complementary evidence-integration framework that can identify evidence gaps, rather than as a replacement for established regulatory risk-assessment approaches. In the present study, TRAEC was used to integrate the available evidence concerning exposure to MNPs, endometrial injury, and adverse pregnancy outcomes and to supplement the evidence base with original in vitro experiments using primary human endometrial stromal cells.

## 2. Materials and Methods

### 2.1. The Framework of Targeted Risk Assessment of Environmental Chemicals (TRAEC)

In this study, the TRAEC 1.1 strategy was employed to conduct a targeted health-risk assessment of evidence relating exposure to MNPs to endometrial function and pregnancy outcomes. TRAEC 1.1 is an enhanced version of TRAEC 1.0 [22].

The TRAEC framework consists of three sequential steps. First, a scientific question is formulated: “What adverse effects of exposure to MNPs on endometrial function and pregnancy outcomes are supported by the available evidence, and what targeted health-risk category is indicated within the TRAEC framework?” Next, evidence was collected through targeted literature searches and laboratory studies across three evidence types: epidemiological studies, in vivo experiments, and in vitro experiments. Finally, the collected evidence was quantitatively evaluated using four complementary dimensions defined in TRAEC 1.1: Reliability Scores, Weight of Concentrations, Risk Intensity, and Correlation Scores. These four dimensions were predefined in TRAEC 1.1 and were applied because they capture complementary aspects of evidence appraisal—methodological reliability, concentration context, effect intensity, and direction of association—and were neither selected nor reweighted specifically for the present dataset. Reliability was assessed using ten criteria specific to each evidence type, with each criterion scored as 0, 0.5, or 1, yielding a total Reliability Score of 0–10. Weight of Concentrations was calculated within each evidence type using the inverse-logarithmic weighting equation. The lowest effect concentration was normalized to 10, and the remaining concentrations were rescaled proportionally, thereby assigning relatively greater weight to evidence reporting effects at lower concentrations. Risk Intensity was scored as 1, 0.8, or 0.4 according to evidence-type-specific criteria. For epidemiological evidence, these scores represented large, moderate, or small absolute effect sizes based on Cohen’s d or Hedges’ g; for in vivo evidence, they represented phenotypic changes with or without molecular or cellular changes, molecular or cellular changes alone, or no detected change; and for in vitro evidence, they represented both cellular-phenotypic and molecular changes, either type of change alone, or no detected change. Correlation was scored as 1, 0, or −1 for a positive, nonsignificant, or negative association, respectively. The score for each evidence item and the Comprehensive Evidence Score (CES) were calculated according to the predefined TRAEC 1.1 equations shown below:

$$ComprehensiveEvidenceScore=\frac{\sum_{1}^{i}\omega_{k}\varepsilon_{k}\varphi r}{i}$$

$$\varepsilon_{k}=\frac{\frac{1}{\mathrm{lg}C_{k}}}{\sum_{1}^{n}\frac{1}{\mathrm{lg}C_{n}}}$$

where *ω*, *ε*, *φ*, and *r* denote the Reliability Scores, Weight of Concentrations, Risk Intensity, and Correlation Scores, respectively; *C* represents the lowest concentration of effects; *n* is the number of evidence items within a single evidence domain; and *i* is the total number of evidence items. The epidemiological, in vivo, and in vitro evidence types were scored separately using their respective criteria and were then integrated at the item level through the CES equation; no additional fixed evidence-type coefficients were applied. In accordance with TRAEC 1.1, the original hESC experiment conducted in the present study was included as one additional in vitro evidence item in the final CES calculation. Following the TRAEC 1.1 procedure, all evidence items were independently scored by two researchers, and the two assessor-specific item-level scores were averaged to obtain the final score for each evidence item. The final CES was then calculated from these averaged item-level scores. A third researcher would be consulted if the two assessor-specific Reliability Scores differed by ≥2 points. Risk Intensity judgments were required to be consistent between assessors. For transparency, the two assessor-specific scoring datasets and corresponding CES values are provided in Supplementary Tables S2 and S3. All calculations were performed using the TRAEC web calculator (https://traec.njmu.edu.cn/; accessed on 19 July 2026).

According to the predefined TRAEC 1.1 thresholds, CES values in the ranges of (0, 4], (4, 8], and (8, 10] correspond to low, moderate, and high risk categories, respectively. Negative CES values are correspondingly interpreted as protective categories of low, moderate, or high magnitude. For the purposes of this study, hazard denotes the capacity of exposure to MNPs to produce adverse effects under specified experimental conditions, whereas risk denotes the likelihood and magnitude of such effects under defined exposure conditions. Accordingly, experimental studies were interpreted primarily as hazard evidence, while the CES was treated as a framework-specific risk classification rather than a direct estimate of real-world human risk. The concentration-weighting term represents a relative adjustment within each evidence type and was not interpreted as a formal quantitative dose–response assessment, a point of departure, or a basis for direct extrapolation to human exposure levels.

### 2.2. Existing Research on the Impact of Exposure to MNPs on Uterine Health

#### 2.2.1. Search Strategy

A systematic search was conducted in the PubMed, Web of Science, Embase, and Scopus databases up to November 2025, focusing on studies examining the associations between “microplastics/nanoplastics” and “uterine health,” “endometriosis,” “uterine fibroids,” and “adverse pregnancy outcomes,” among other related topics. The detailed search strategies and keywords are provided in Supplementary Table S1. In addition, the reference lists of included studies were manually searched to supplement the relevant literature.

#### 2.2.2. Inclusion and Exclusion Criteria

Inclusion criteria:

(a) Study types include cohort studies, cross-sectional studies, case–control studies, in vivo animal experiments, or in vitro cell experiments;
(b) The study examined the occurrence, distribution, association, or biological effects of MNPs in relation to female reproductive tissues, uterine or endometrial function, implantation, placental function, pregnancy outcomes, or fetal/offspring outcomes in human populations, animal models, or relevant in vitro models;
(c) Provides quantitative effect indicators or specific toxicity detection data;
(d) Clearly defines the characteristics of exposure to MNPs (e.g., polymer type, particle size, etc.);
(e) The study included at least one outcome relevant to female reproductive tissues, uterine or endometrial health, implantation, placental function, pregnancy, or fetal/offspring development.

Exclusion criteria:

(a) Duplicate publications, non-original research, or literature that does not meet the inclusion criteria;
(b) Studies with incomplete information or missing data that cannot be extracted;
(c) Studies not relevant to MNP exposure and female reproductive tissues, uterine or endometrial health, implantation, placental function, pregnancy outcomes, or fetal/offspring outcomes;
(d) Literature for which the full text or key data cannot be obtained.

The full-text information of the literature was independently extracted by two researchers, including the title, authors, study period, study design, region, population characteristics (or experimental subject information), details of exposure to MNPs (polymer type, particle size, concentration, and exposure route), outcome indicators, and detection methods. Discrepancies were resolved through group discussion, and original authors were contacted to supplement any missing key data.

### 2.3. PS-NPs and Exposure Design

Polystyrene nanoplastics (PS-NPs; manufacturer-specified nominal diameter, 50 nm; Cat. No. DS50) were obtained from Huge Biotechnology (Shanghai, China). The stock suspension was stored in a sealed container at 4 °C and sonicated for 5–10 min before each use. Working suspensions were freshly prepared in complete culture medium immediately before exposure. Concentrations of 50, 100, and 200 μg/mL were selected on the basis of preliminary range-finding experiments conducted by our group and concentration ranges reported in relevant in vitro studies. The preliminary data were used solely to guide concentration selection and are not presented in the current manuscript. hESCs were exposed to 0, 50, 100, or 200 μg/mL PS-NPs for 24 h.

### 2.4. Isolation and Culture of Human Primary Endometrial Stromal Cells

Endometrial tissue was treated with collagenase I (Solarbio Science & Technology, Beijing, China) to obtain human primary endometrial stromal cells (hESCs). The isolated hESCs were cultured in a DMEM/F12 medium, supplemented with 5% fetal bovine serum and 1% penicillin–streptomycin (all from Sigma-Aldrich, Inc., St. Louis, MO, USA), and cultured in a cell incubator at 37 °C and 5% carbon dioxide.

### 2.5. Detection of Reactive Oxygen Species

The hESCs treated with different concentrations of PS-NPs were observed and photographed using a Ti2-U inverted microscope equipped with a Digital Sight 10 camera and NIS-Elements D software (version 6.10.00, Build 2017; Nikon Corporation, Tokyo, Japan). The reactive oxygen species (ROS) kit (Solarbio Science & Technology, Beijing, China) was used to detect intracellular reactive oxygen species levels. The hESCs treated with different concentrations of PS-NPs were washed three times with PBS, then DCFH-DA solution (1 mL of 10 μM) was added and incubated at 37 °C for 30 min in the dark. After washing three times with PBS, the cells were observed and photographed using the same microscope in fluorescence mode.

### 2.6. Mitochondrial Membrane Potential Detection (JC-1)

The mitochondrial membrane potential detection kit (JC-1) (Solarbio Science & Technology, Beijing, China) was used to detect the level of mitochondrial membrane potential of hESCs treated with PS-NPs. The hESCs treated with PS-NPs were washed three times with PBS, incubated with JC-1 staining working solution at 37 °C for 20 min in the dark, and then washed twice with JC-1 staining buffer to remove unbound dyes. The red and green fluorescence brightness was detected by fluorescence microscopy.

### 2.7. Cell Apoptosis Assay

Annexin V-FITC/PI Apoptosis Detection Kit (Multi Sciences, Hangzhou, China) was used to detect the level of apoptosis according to the standard process provided with the kit. The hESCs were treated with different concentrations of PS-NPs, washed with PBS, digested with trypsin, and centrifuged. The precipitate was suspended in the binding buffer and incubated with 5 μL of Annexin V-FITC and 10 μL of propidium iodide (PI) at room temperature and in the dark for 5 min, followed by quantitative analysis of apoptosis by flow cytometry.

### 2.8. Western Blot

Protein samples were electrophoresed on 8% or 10% polyacrylamide gels and transferred to a 0.22 μm PVDF membranes (Immobilon Transfer Membrane; MilliporeSigma, Burlington, MA, USA). The membrane was blocked in TBST buffer containing 5% skim milk powder for 2 h at room temperature and then incubated with different antibodies, GAPDH, BCL2, BAX, ITGB3, FOXO1, and HOXA10 (Proteintech Group, Inc., Wuhan, China), at 4 °C overnight. Then it was incubated with HRP-labeled secondary antibody (Proteintech Group, Inc., Wuhan, China) at room temperature for 2 h and then immersed in ECL luminescent solution (Coolaber Technology, Beijing, China) and exposed. Finally, ImageJ software (version 1.53a; National Institutes of Health, Bethesda, MD, USA) was used to analyze the gray level of the band.

### 2.9. Statistical Analysis

All quantitative experiments were performed with at least three independent biological replicates, and the data are presented as the mean ± standard deviation (SD). Normality was assessed using the Shapiro–Wilk test, and homogeneity of variance was evaluated using Levene’s test. Comparisons among multiple groups were performed using one-way analysis of variance (ANOVA). Fisher’s least significant difference (LSD) post hoc test was used when the assumption of homogeneity of variance was satisfied, whereas Tamhane’s T2 test was used when variances were unequal. Statistical analyses were performed using SPSS version 25.0 (IBM Corp., Armonk, NY, USA). All tests were two-sided, and a *p* value < 0.05 was considered statistically significant.

## 3. Results

### 3.1. Problem Statement

The potential effects of exposure to MNPs on female reproductive health have raised increasing concern. Based on the TRAEC risk assessment framework, we formulated the following scientific question: ‘What adverse effects of exposure to MNPs on endometrial function and pregnancy outcomes are supported by the available evidence, and what risk category is indicated by the integrated evidence within the TRAEC framework?’ Problem formulation constitutes the foundational step in TRAEC-based risk assessment.

### 3.2. Evidence Collection and TRAEC Results

**Figure 1:**
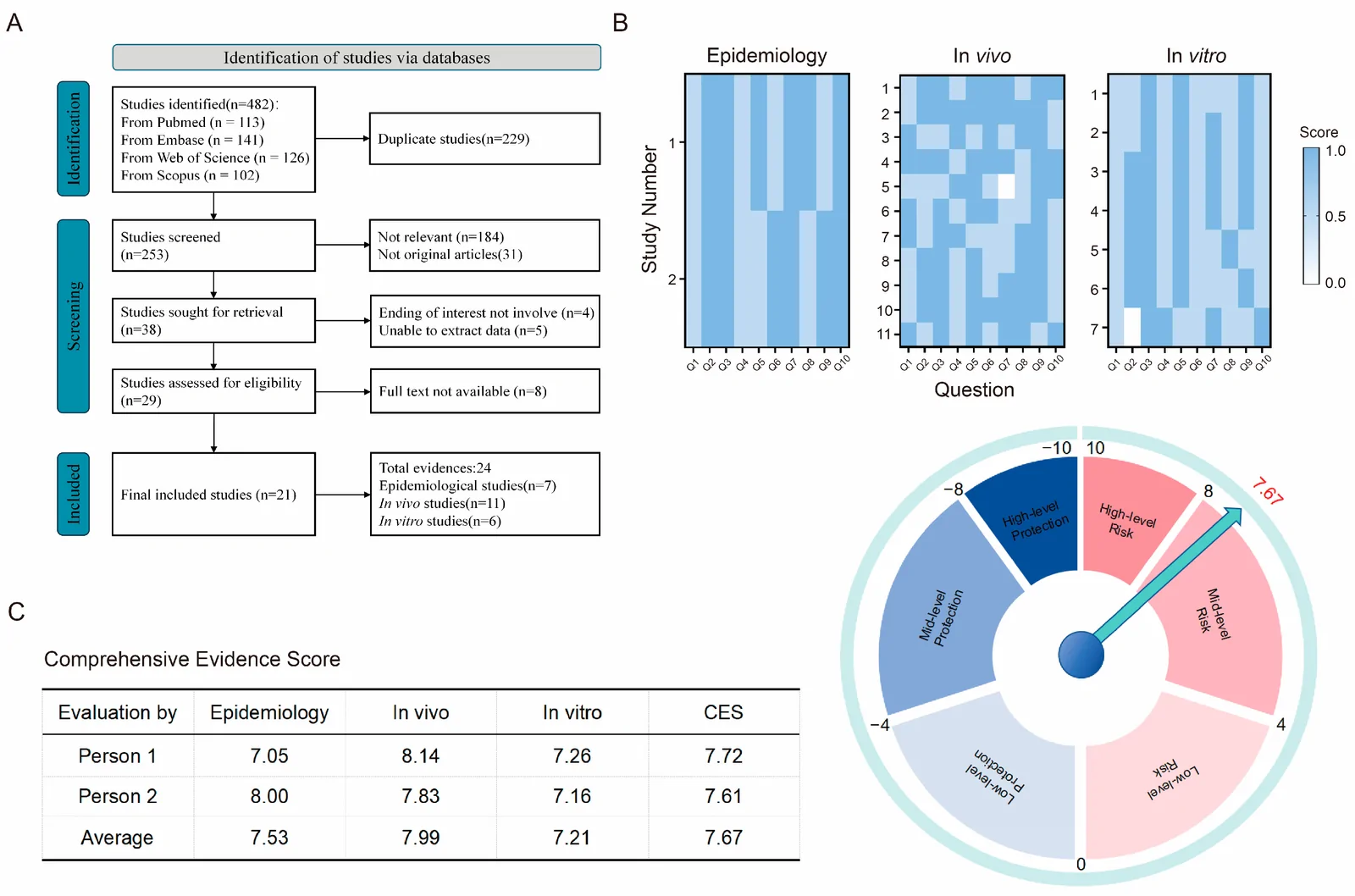
Study selection and evidence scoring under the TRAEC framework. (**A**) Study selection according to the TRAEC methodology. (**B**) Reliability scores for epidemiological, in vivo, and in vitro studies evidence items. (**C**) Comprehensive Evidence Score (CES). In the dial chart, the arrow indicates the final average CES (7.67); the pink and blue sectors represent risk and protection, respectively, with darker shades indicating higher levels. The 24 literature-derived evidence entries were supplemented with one original in vitro evidence item for the CES calculation.

A total of 482 records were identified. After removal of 229 duplicates, 253 records were screened; 184 irrelevant records and 31 non-original articles were excluded, leaving 38 records for further assessment. Of these, 4 did not report outcomes of interest and 5 lacked extractable data, leaving 29 records for eligibility assessment. Eight of these records were subsequently excluded because their full texts were unavailable. Consequently, 21 unique studies were included in the TRAEC assessment (Figure 1A). Because some studies contributed to more than one evidence domain, these 21 studies generated 24 domain-specific evidence entries: 7 epidemiological, 11 in vivo, and 6 in vitro. The original hESC experiment conducted in the present study was included as one additional in vitro evidence item, resulting in 25 evidence items for the final CES calculation: 7 epidemiological, 11 in vivo, and 7 in vitro. Table 1, Table 2 and Table 3 summarize the literature-derived evidence, whereas the original hESC experiment is presented in Section 3.3. All 21 studies were published between 2020 and 2025, of which 12 were published in 2025 and 6 in 2024.

Among the seven epidemiological studies included, the research primarily focused on the occurrence of MNPs in endometrial, placental, and other female reproductive samples, as well as their associations with uterine conditions and pregnancy outcomes. There were four cross-sectional studies and three case–control studies. Four of these studies were conducted in China, one in the United Kingdom, one in the United States, and one in Iran. Among these seven studies, two studies reported associations between placental MNP burden and adverse pregnancy outcomes. One study found higher placental MNP levels in preterm pregnancies, whereas another reported inverse associations between placental MP burden and neonatal anthropometric measurements. Another study reported higher MP abundance and significantly higher PS concentrations in endometrial polyps than in normal endometrium. The detection methods for MPs mainly included micro-Raman spectroscopy, micro-Fourier transform infrared spectroscopy, pyrolysis-gas chromatography/mass spectrometry (Py-GC/MS), and laser direct infrared spectroscopy (LDIR). The concentration units of MPs in the tested samples were not standardized, with units such as particles/100 mg, μg/g, particles/L, and particles/g, making direct comparisons between studies impossible. Therefore, future studies should harmonize reporting metrics, including tissue-mass normalization, particle abundance, polymer-specific mass, particle-size distributions, and procedural blank correction, to improve comparability and validation across studies.

A total of 11 in vivo studies evaluated the effects of exposure to MNPs on endometrial function, adverse pregnancy outcomes, and offspring health. In the included studies, ten studies reported alterations in uterine, endometrial, decidual, endocrine, or uterine-vascular endpoints after experimental exposure, including endometrial thinning, adhesions, inflammatory infiltration, reduced uterine gland numbers, altered epithelial or mitochondrial ultrastructure, impaired decidualization, decreased uterine arteriole number or diameter, uterine endothelial dysfunction, altered reproductive hormone signaling, and reduced receptivity-related markers. Six studies reported impaired implantation, reduced litter size, or increased embryo resorption following experimental exposure to MNPs. Five studies assessed fetal or offspring outcomes: two reported reduced fetal weight; one identified altered metabolomic profiles in female offspring; one reported an altered offspring sex ratio together with a reduced litter size, and one found no significant changes in the measured litter or fetal outcomes.

A total of 6 in vitro studies were included. One of the studies was related to placental trophoblast cells, using human primary villous trophoblast cells and chorionic-villus explants. The other 5 studies were related to the endometrium, using human primary endometrial stromal cells, human primary endometrial epithelial cells, mouse endometrial epithelial cell lines, a scaffold-based three-dimensional human endometrial co-culture model, and human endometrial organoids. Across these models, exposure to PS particles produced concentration- and size-dependent changes in cellular uptake, viability, epithelial-barrier integrity, apoptosis, migration, oxidative stress, inflammatory signaling, endocrine function, and pro-fibrotic responses.

The TRAEC Comprehensive Evidence Score (CES) integrated evidence across the epidemiological, in vivo, and in vitro domains. Application of the TRAEC 1.1 scoring procedure yielded assessor-specific CES values of 7.72 and 7.61. Following the predefined dual-assessor procedure, the final CES calculated from the averaged item-level scores was 7.67. This value falls within the predefined TRAEC moderate-risk interval of (4, 8] (Supplementary Tables S2 and S3; Figure 1B,C).

### 3.3. Effects of Exposure to PS-NPs on Cellular Injury and Endometrial Receptivity Markers in hESCs

Based on the TRAEC framework, we conducted additional experiments to evaluate the effects of exposure to PS-NPs on primary human endometrial stromal cells (hESCs). Multiple polymer types were identified in the included epidemiological studies, whereas polystyrene was the predominant polymer used in the included in vivo and in vitro experimental studies (Table 2 and Table 3). Therefore, PS-NPs were selected for the present experiments.

**Figure 2:**
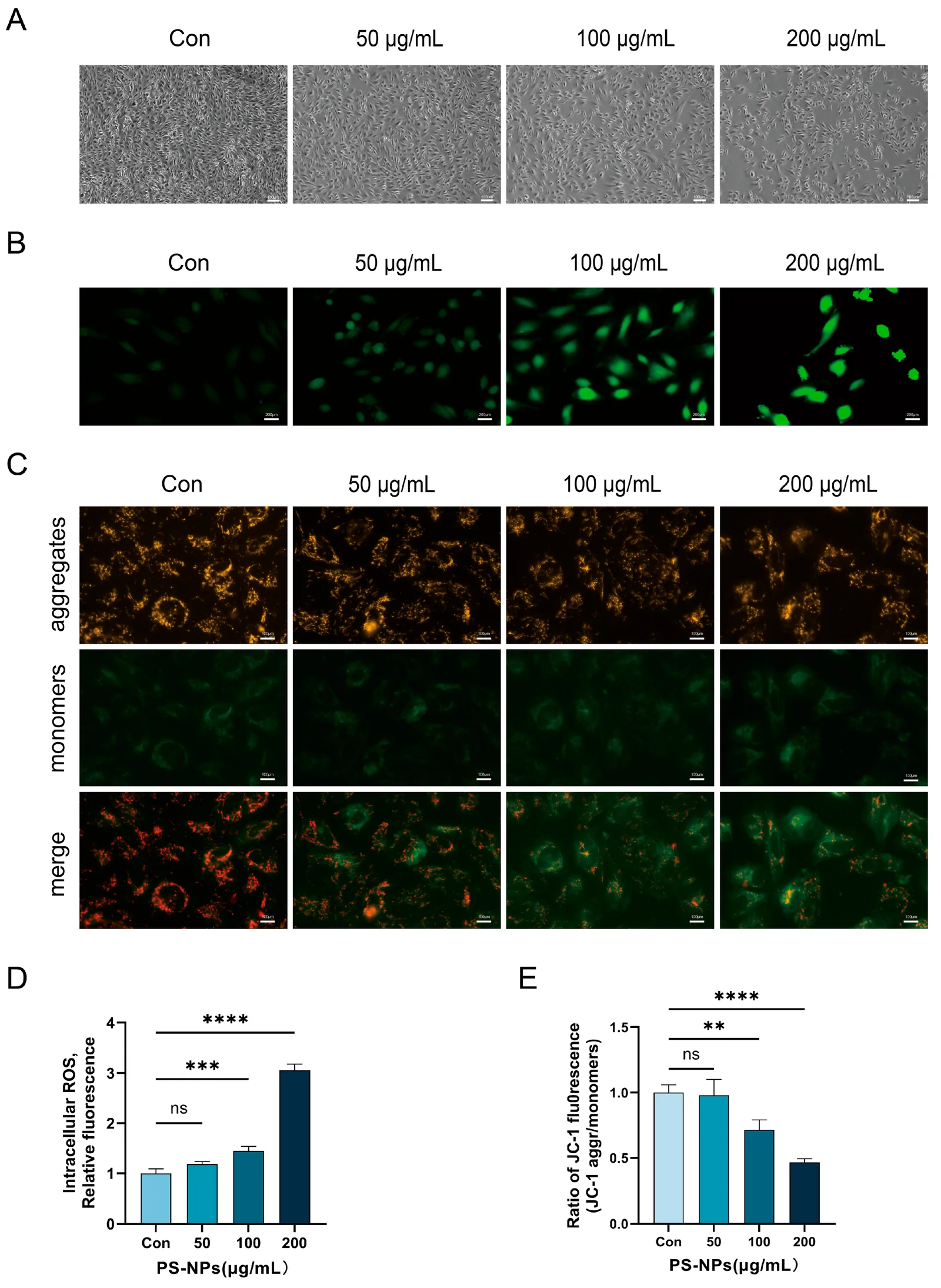
Morphological changes, intracellular ROS accumulation, and reduced mitochondrial membrane potential in hESCs after exposure to PS-NPs. hESCs were treated with 0, 50, 100, and 200 μg/mL PS-NPs for 24 h. (**A**) Morphological changes in hESCs; scale bar = 100 μm. (**B**,**D**) Intracellular ROS production detected using the DCFH-DA probe; scale bar = 200 μm. (**C**,**E**) Changes in mitochondrial membrane potential (MMP) detected via the JC-1 probe. JC-1 monomers are represented by green fluorescence, while JC-1 aggregates are represented by red fluorescence; scale bar = 100 μm. ns, not significant; ** *p* < 0.01, *** *p* < 0.001, and **** *p* < 0.0001 vs. the control group.

First, we examined the morphological changes in hESCs after exposure to PS-NPs. As shown in Figure 2A, with increasing concentrations of PS-NPs, the cell number gradually decreased, and the spindle-shaped morphology became increasingly irregular. Subsequently, intracellular ROS levels were assessed. The results showed that exposure to 50 μg/mL PS-NPs for 24 h did not significantly alter intracellular ROS levels. However, when the concentration of PS-NPs reached 100 μg/mL, intracellular ROS levels were significantly higher than those in the control group (*p* < 0.01) and increased with increasing concentrations of PS-NPs (Figure 2B,D). Further assessment of mitochondrial membrane potential was performed using JC-1 staining (Figure 2C,E). The intensity of intracellular green fluorescence gradually increased with increasing PS-NP concentrations, consistent with progressive mitochondrial membrane depolarization.

**Figure 3:**
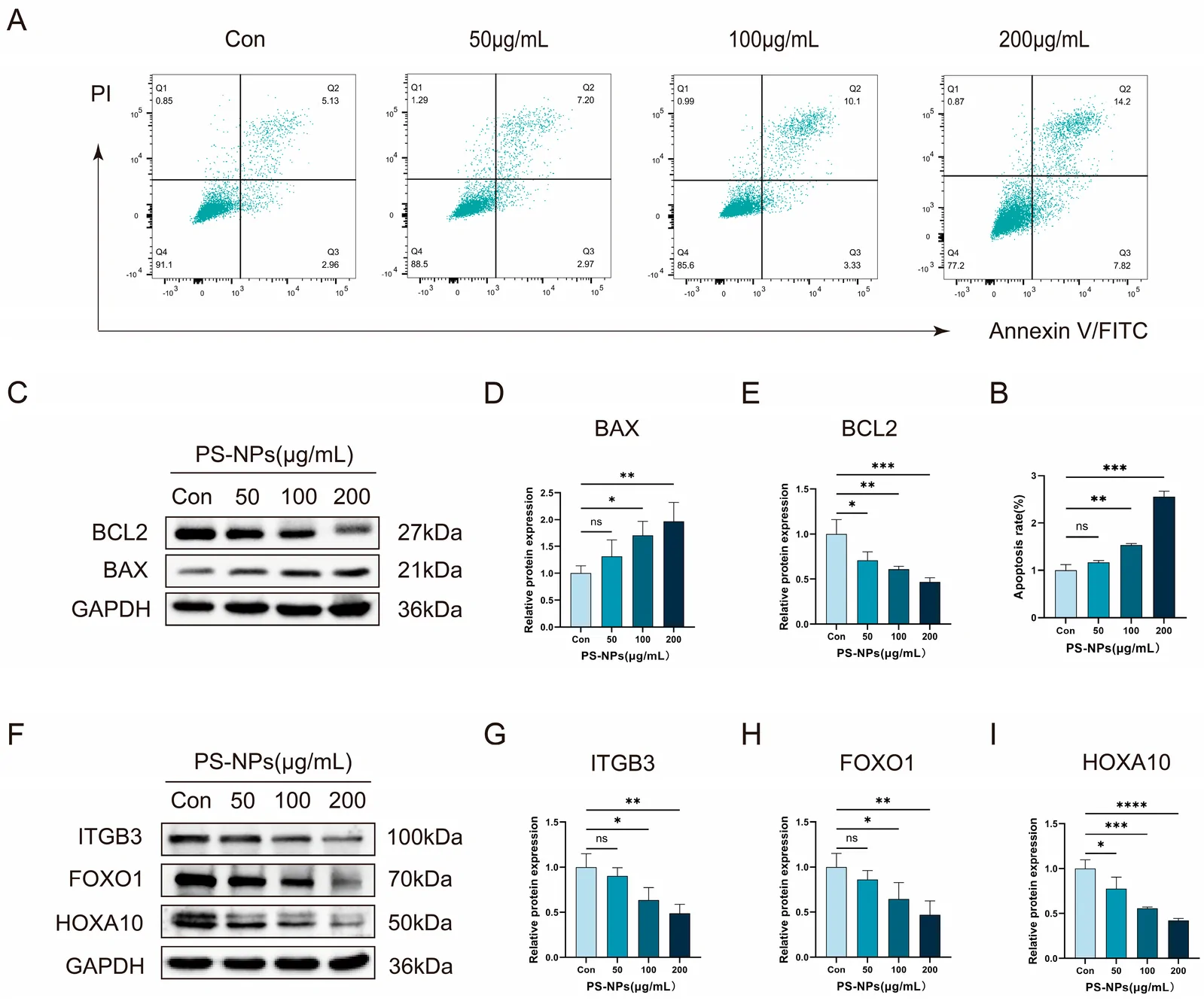
Apoptosis and endometrial receptivity-related protein expression in hESCs after exposure to PS-NPs. hESCs were treated with 0, 50, 100, and 200 μg/mL PS-NPs for 24 h. (**A**,**B**) Apoptosis levels detected by flow cytometry. (**C**–**E**) Protein expression levels of BAX and BCL2 determined by Western blot. (**F**–**I**) Protein expression levels of FOXO1, HOXA10, and ITGB3 determined by Western blot. ns: not significant; * *p* < 0.05, ** *p* < 0.01, *** *p* < 0.001, and **** *p* < 0.0001 vs. the control group.

Because loss of mitochondrial membrane potential is associated with early apoptotic changes, apoptosis was further assessed by flow cytometry and Western blotting. The results of flow cytometry (Figure 3A,B) showed that the percentages of early apoptotic cells were 2.96%, 2.97%, 3.33%, and 7.82%, whereas the percentages of late apoptotic cells were 5.13%, 7.20%, 10.10%, and 14.20% after exposure to 0, 50, 100, and 200 μg/mL PS-NPs, respectively. Both early and late apoptosis increased with increasing concentrations of PS-NPs, and total apoptosis was significantly higher after exposure to 100 μg/mL PS-NPs than in the control group (*p* < 0.01). The pro-apoptotic effect of PS-NPs was further verified by detecting the expression of key apoptosis regulatory proteins. The results showed that exposure to PS-NPs significantly increased the expression of the pro-apoptotic protein BAX and decreased the expression of the anti-apoptotic protein BCL2 (Figure 3C–E). To evaluate endometrial receptivity-related molecular changes, we measured the protein expression of HOXA10, FOXO1, and ITGB3. The expression of all three proteins decreased significantly with increasing concentrations of PS-NPs (Figure 3F–I), demonstrating concentration-dependent downregulation of endometrial receptivity-related proteins.

## 4. Discussion

MNPs are widespread pollutants whose biological effects vary with polymer type, size, shape, and surface properties [40,41]. In this study, TRAEC was used to integrate epidemiological, in vivo, and in vitro evidence related to endometrial function and pregnancy outcomes.

Real-world exposure to MNPs may occur through ingestion of contaminated food and drinking water and inhalation of airborne particles [40,41]. MPs have also been detected in the human endometrium. Sun et al. identified 13 polymer types (20–500 μm) in human endometrial samples and reported potential associations with lifestyle factors [15]. Occupational exposure was not specifically evaluated in the included reproductive studies and remains an important evidence gap. Recent studies have detected MNPs in human follicular fluid and MPs in semen. Higher follicular-fluid PA66 and PVC concentrations were associated with greater odds of diminished ovarian reserve in a recent case–control study [42]. A semen study detected multiple MP polymers and observed a nonsignificant trend toward lower progressive sperm motility in PET-positive samples [43]. These findings document reproductive exposure but do not yet establish clinically meaningful effects on fertility.

Experimental evidence suggests that MNPs may affect female reproductive function through several interacting, but not yet unified, pathways [44]. Complementary cross-species studies in male mussels reported oxidative and metabolic stress, sperm-chromatin disorganization, and altered protamine-like protein-DNA interactions [45,46]. In our hESC model, PS-NPs increased intracellular ROS, reduced mitochondrial membrane potential, promoted apoptosis, and decreased HOXA10, FOXO1, and ITGB3 expression under the tested conditions. These concurrent changes are consistent with, but do not prove, a mechanistic sequence linking oxidative and mitochondrial injury to apoptosis and downregulation of receptivity-related proteins. Complementary experimental studies provided pathway-level support for several of these responses. In mouse endometrial epithelial cells, pharmacological inhibition of TLR4 or NOX2 attenuated PS-MP-induced oxidative stress, downstream Notch/TGF-β signaling, and pro-fibrotic changes [28]. In mice, JNK inhibition partially restored implantation, decidualization, and stromal-cell proliferation after PS-NP exposure, whereas another study reported activation of uterine TLR4/MyD88/NF-κB and NLRP3 signaling together with reduced implantation and receptivity-related markers [29,35]. Peri-implantation exposure to PS-MPs also altered decidual natural killer cells, macrophage polarization, placental CD4^+^ T cells, and cytokine secretion, together with reduced uterine arterioles and increased embryo resorption [34]. Because successful implantation requires a temporally balanced immune environment at the maternal–fetal interface [11], these findings support biologically plausible inflammatory, immune, and vascular links to impaired implantation. However, the relevant immune-cell and cytokine endpoints were not measured in our hESC experiments, and their relevance to humans remains unconfirmed. Endocrine disruption may provide a parallel pathway. One rat study reported reduced StAR expression in luteal cells and lower serum progesterone [30], whereas exposure of primary human villous cytotrophoblasts to PS-NPs reduced hCG secretion and elicited particle-size- and concentration-dependent pro-inflammatory responses without significantly altering VCT-to-ST fusion [39].

Beyond early reproductive events, evidence linking exposure to MNPs to adverse pregnancy outcomes remains limited and heterogeneous. Experimental work showed that gestational exposure to MNPs impaired placental trophoblast syncytialization and activated the PERK/eIF2α/ATF4 signaling pathway; pharmacological PERK inhibition partially restored syncytialization in BeWo cells [47], whereas observational data reported higher placental MP burdens in pregnancies complicated by fetal growth restriction and inverse associations with selected neonatal anthropometric measurements [27]. MPs have also been detected on the maternal and fetal sides of human placentas and in the chorioamniotic membranes [48], and an ex vivo human placental perfusion model demonstrated size-dependent passage of fluorescent PS beads up to 240 nm without altering the assessed viability or function of the perfused placental tissue [49]. However, placental detection alone does not establish transplacental transfer or quantify fetal exposure, and the perfusion findings were obtained under controlled experimental conditions. Overall, the available evidence supports biologically plausible links to impaired decidualization, implantation, placental function, embryo survival, and fetal growth, but it does not establish a unified causal mechanism or quantify real-world human reproductive risk.

In vitro models provide controlled mechanistic evidence but cannot reproduce whole-body absorption, biodistribution, clearance, or interactions among reproductive, immune, vascular, endocrine, placental, and embryonic compartments, nor can they directly assess implantation or fetal development. More broadly, the experimental evidence is dominated by rodent and in vitro models, often using relatively high doses and short exposure durations. Heterogeneous dose metrics prevent direct comparison with human tissue burdens; most experiments examined polystyrene particles under controlled laboratory conditions rather than real-world mixtures; and the available human studies were observational and generally involved limited sample sizes. Accordingly, experimental studies mainly inform hazard identification, biological plausibility, and model-specific adverse effects, whereas human studies establish tissue occurrence and preliminary associations. Together, these limitations constrain causal interpretation and quantitative risk estimation.

Although experimental evidence was dominated by polystyrene, epidemiological studies identified polyethylene, polypropylene, polyvinyl chloride, polyethylene terephthalate, and mixed polymer profiles in human reproductive tissues [15,24,25]. TRAEC is not intrinsically polymer-specific and could, in principle, be applied to individual polymers or mixtures when sufficiently detailed evidence is available [9,22]. However, the present CES should not be interpreted as polymer- or mixture-specific because particle properties, additives, weathering, and co-contaminants may modify biological effects [40]. Future assessments should therefore stratify evidence by polymer and particle characteristics.

The environmental relevance of the experimental exposure levels remains uncertain. One included mouse study selected 50 mg/kg/day with reference to published estimates of human MP ingestion and a mouse-equivalent dose range calculated using body-surface-area scaling [29,50,51,52]. However, the ingestion estimates were derived from heterogeneous data and conservative assumptions, and the allometric conversion does not directly account for particle-specific absorption, biodistribution, clearance, or uterine deposition [51,52]. Human tissue burdens cannot be directly compared with administered animal doses or nominal in vitro concentrations. Accordingly, the experimental concentrations, including 50–200 μg/mL in our hESC experiments, should be interpreted as hazard-identification conditions rather than as quantitatively equivalent to realistic human uterine exposure.

TRAEC provides a transparent framework for integrating epidemiological, in vivo, and in vitro evidence using predefined criteria [22,23]. The final CES of 7.67 falls within the framework’s moderate-risk category, but the evidence streams contribute differently: experimental studies mainly support hazard identification, biological plausibility, and model-specific adverse effects, whereas epidemiological studies report MNP occurrence and preliminary associations without standardized exposure estimates or quantitative links between external exposure, tissue dose, and adverse outcomes. Initial concordance with ToxRTool, SciRAP, OHAT RoB, and IRIS-based evaluations, together with subsequent applications, supports the feasibility of TRAEC [9,22,23], but its external validation remains preliminary. The CES therefore summarizes the current evidence but does not replace formal dose–response or exposure assessment or quantify real-world human risk. TRAEC should support evidence synthesis and gap identification rather than serve as a stand-alone basis for regulatory decision-making.

## 5. Conclusions

In this study, TRAEC 1.1 was used to integrate epidemiological, in vivo, and in vitro evidence concerning exposure to MNPs, endometrial injury, and adverse pregnancy outcomes. The final CES of 7.67 falls within the moderate-risk category of the predefined TRAEC framework and should be considered a preliminary classification based on the currently available evidence. Our hESC experiments provide mechanistic hazard evidence that exposure to PS-NPs increased intracellular ROS, reduced mitochondrial membrane potential, promoted apoptosis, and decreased the expression of key endometrial receptivity proteins such as HOXA10, FOXO1, and ITGB3 under the tested conditions. However, the experimental evidence is dominated by in vitro systems and rodent models; many studies used relatively high exposure levels, and most examined polystyrene particles rather than the heterogeneous mixtures encountered under real-world conditions. These limitations reduce confidence in direct extrapolation to environmentally relevant human exposure conditions. Well-designed prospective human studies and experimental models incorporating environmentally informed exposure ranges, diverse polymers, and more realistic particle characteristics are required to refine this classification.

## Figures and Tables

**Table 1:** The contextual details of included epidemiological studies.

ID	Study	Location	Study Design	Study Population/ Samples	Particle Type; Size	Particle Concentration/Abundance	Uterine/Reproductive Findings	Detection Techniques	Key Findings
1	Qin et al. 2024 [17]	China	Cross-sectional descriptive study	Endometrial tissue samples from 22 women with recurrent spontaneous abortion undergoing hysteroscopic surgery	Main detected polymers included PA, PU, PET, PP, PS, and PE; approximately 2–200 μm	MPs were detected in all 22 endometrial samples; accurate particle abundance per unit tissue mass was not reported	MPs were detected and characterized in endometrial samples from women with recurrent spontaneous abortion; no control group or human reproductive outcome comparison was included	Raman microspectroscopy and FTIR imaging using an Agilent 8700 LDIR system	MPs of diverse polymer types and sizes were detected in all endometrial samples from women with recurrent spontaneous abortion; their relationship with pregnancy loss could not be determined
2	Sun et al. 2024 [15]	China	Cross-sectional descriptive study	Endometrial tissue samples from 20 women undergoing gynecological surgery	Thirteen MP types; 20–500 μm, with 88.35% measuring 20–100 μm	Total MP abundance ranged from 0 to 117 particles/100 mg tissue, with a median of 21 particles/100 mg	MP abundance was evaluated in relation to age, BMI, and lifestyle factors; no uterine pathology or reproductive outcomes were assessed	Laser direct infrared spectroscopy using an Agilent 8700 LDIR system	Thirteen MP types were detected in human endometrium; selected beverage habits and chewing gum use were associated with higher MP abundance
3	He et al. 2025 [16]	China	Case–control study	Endometrial polyps from 16 women and normal endometrial samples from 14 women	Py-GC/MS quantified PS, PE, and PVC; LDIR identified 13 MP types in polyp samples, predominantly PMMA; 20–500 μm, with 95.94% measuring 20–100 μm	Polyp vs. normal endometrium: PS, 13.66 ± 2.00 vs. 7.132 ± 0.78 μg/g (*p* = 0.0009); PE, 94.81 ± 10.67 vs. 69.29 ± 6.93 μg/g (*p* = 0.0621); PVC, 67.67 ± 11.02 vs. 56.35 ± 6.90 μg/g (*p* = 0.0595)	Overall MP abundance and PS concentrations were higher in endometrial polyps than in normal endometrium; causal direction was not assessed	Py-GC/MS for polymer-specific mass quantification and LDIR for particle-level polymer identification and size characterization	Endometrial polyps showed higher overall MP abundance and significantly higher PS concentrations than normal endometrium; causal direction could not be determined
4	Jochum et al. 2025 [24]	United States	Case–control study	Placentae from 158 cesarean deliveries: 87 term (≥37 weeks) and 71 preterm (<37 weeks)	Twelve polymers quantified, including PE, SBR, PVC, PP, N66, PET, N6, PMMA, ABS, PU, PC, and PS; particle size was not resolved by Py-GC/MS	Total MNP concentrations were 224.7 ± 180.7 μg/g in preterm vs. 175.5 ± 137.9 μg/g in term placentae; PVC, PET, PU, and PC were significantly elevated in preterm placentae	Placental MNP concentrations were evaluated in relation to preterm birth, gestational age, birth weight, and maternal clinical characteristics; PVC and PC were independently associated with preterm birth in adjusted logistic regression	Py-GC/MS for polymer-specific mass quantification, with correlation and multivariable logistic regression analyses	Placental MNP concentrations were 28% higher in preterm deliveries; selected polymers were associated with preterm birth, gestational age, and birth weight
5	Dong et al. 2024 [25]	China	Cross-sectional descriptive study	Tissue samples from 60 women: 20 with adenomyosis, 20 with ovarian endometriotic cysts, and 20 with fallopian-tube disorders	Eleven polymers, mainly PE (31%), PP (22%), and PE-co-PP (11%); particle length ranged from 4.51 to 32.93 μm, with 70% <20 μm	MPs were detected in 43/60 samples; mean abundance was 1.50 ± 1.20 particles/g, or 1.40 ± 1.11 particles/g after blank correction	MP abundance in adenomyosis samples correlated with age and BMI; no significant corresponding correlations were found in ovarian cyst or fallopian-tube samples, and fertility outcomes were not assessed	Micro-Raman spectroscopy following alkaline digestion and oxidative treatment, with procedural blank correction	MPs were detected in 43 of 60 diseased reproductive-tissue samples; PE and PP predominated, and MP abundance in adenomyosis samples correlated with age and BMI
6	Rotchell et al. 2024 [26]	United Kingdom	Comparative cross-sectional study	Urine samples from 19 healthy donors and 19 participants with endometriosis, with 15 procedural blanks analyzed in parallel	Twenty-two polymer types overall: 18 in healthy donors and 16 in endometriosis participants; dominant polymers were PE, PS, resins, and PP in healthy donors and PTFE and PE	Unadjusted mean abundance was 2589 ± 2931 MP/L in healthy donors and 4724 ± 9710 MP/L in endometriosis participants, with no significant group difference (*p* = 0.38); procedural blanks contained 17 ± 18 particles/sample	Total urinary MP abundance did not differ significantly between groups, although polymer profiles and particle-size characteristics differed; fertility and uterine outcomes were not assessed	μFTIR spectroscopy, with SEM-EDX validation of a particle subset and extensive procedural blank analysis	MPs were detected in urine from both groups; total abundance did not differ significantly, although polymer profiles differed, underscoring the importance of sampling and blank controls
7	Amereh et al. 2022 [27]	Iran	Case–control study	Placentae from 43 pregnancies: 13 with intrauterine growth restriction and 30 with normal fetal growth	IUGR placentae: PE (43.0%), PS (36.4%), PET (14.9%), and PP (5.6%); control placentae: PE (66.7%) and PS (33.3%); particle sizes were 2.9–34.5 μm in IUGR and 7.3–27.6 μm in controls	A total of 302 particles were detected in IUGR placentae vs. 6 particles in controls; MPs were detected in all 13 IUGR placentae and only a minority of controls	Higher placental MP burden was inversely associated with birth weight, birth length, head circumference, and 1-min Apgar score	Digital microscopy followed by Raman microspectroscopy using a 785-nm laser	MPs were detected more frequently and at higher abundance in IUGR placentae; placental MP burden was inversely associated with neonatal anthropometric measurements and 1-min Apgar score

**Table 2:** The contextual details of included in vivo studies.

ID	Study	Location	Species/Strain	Age/ Stage	Sample Size	Particle Type; Size	Exposure Mode	Pregnancy/Offspring Outcomes	Reproductive-Tissue Morphological Outcomes	Reproductive/Mechanistic Findings	Detection Techniques	Conclusion
1	Wu et al. 2022 [28]	China	SPF mice (female; strain not specified)	6 weeks old	12 total; 6/group	PS-MPs; 5–10 μm	Drinking water containing 100 mg/L PS-MPs for 42 days	Not measured	Uneven and thinned endometrium; epithelial injury, endometrial adhesions, narrowed uterine cavity, severe uterine-wall vacuolation, reduced endometrial gland number, collagen deposition, and fibrotic lesions	Increased uterine ER, PR, and LHR expression; activation of TLR4/NOX2, oxidative-stress, Notch, and TGF-β signaling; increased fibrotic mediators and collagen proteins	H&E, Masson’s trichrome, and Sirius red staining; IHC; IF; qRT-PCR; Western blotting; 8-OHdG and oxidative-stress assays	In mice, exposure to PS-MPs induced endometrial injury and uterine fibrosis accompanied by activation of TLR4/NOX2, oxidative-stress, Notch, and TGF-β signaling. Pathway-inhibitor experiments supporting these mechanisms were performed in cultured mouse endometrial epithelial cells
2	Sun et al. 2025 [29]	China	C57BL/6 mice (female)	3 weeks old; acclimated for 1 week before exposure	60 total; 30/group overall, with endpoint-specific sample sizes of 3–8/group	Fluorescent PS-MPs; 1 μm	Oral gavage at 50 mg/kg/day for 14 days	Altered blood metabolomic profiles in 6-week-old female offspring; 68 differential metabolites overlapped between maternal and offspring samples	Uterine PS-MP deposition and epithelial internalization; abnormal apical microvilli and mitochondrial ultrastructure with reduced or absent cristae	Reduced implantation sites and uterine receptivity markers, including IHH, AREG, HOXA10, LIF, and p-STAT3; abnormal uterine epithelial proliferation and cell death; activation of TLR4/MyD88/NF-κB and NLRP3/caspase-1/GSDMD inflammatory signaling; altered maternal metabolomic profiles	Fluorescence imaging; H&E; TEM; IF; qRT-PCR; Western blotting; transcriptomic sequencing; LC-MS metabolomics; ELISA	In mice, exposure to PS-MPs reduced implantation and uterine receptivity-related markers, induced uterine inflammatory signaling, and was accompanied by distinct maternal and female-offspring blood metabolomic profiles
3	Valencise et al. 2025 [30]	Brazil	Wistar rats (female)	Adult; treatment initiated at postnatal day 110	10/group; 5/group for tissue endpoints and 5/group for fertility assessment	PS-NPs; 500 nm	Oral gavage at 0.015 mg/day, approximately 0.1 mg/kg/day, for 25 consecutive days	Pregnancy rate was numerically lower but not statistically different; implantation-related fertility measures, litter size, fetal and placental weights, and offspring sex ratio were unchanged	Enlarged uterine lumen and blood vessels with inflammatory infiltration; more endometrial glands were observed, but the quantitative increase was not statistically significant; uterine-layer thickness and ovarian morphology were unchanged	Altered estrous-phase distribution, with reduced proestrus and increased diestrus; reduced StAR immunostaining in luteal cells and decreased serum progesterone; serum estradiol was unchanged; uterine and pituitary weights increased, whereas thyroid weight decreased	H&E; uterine morphometry; IHC; ELISA; estrous-cycle monitoring; sexual-behavior and fertility assessment	In adult rats, exposure to PS-NPs altered estrous cyclicity and selected endocrine endpoints, including reduced luteal-cell StAR expression and serum progesterone, without significantly altering the measured fertility outcomes
4	Qin et al. 2024 [17]	China	C57BL/6 mice (female)	6–8 weeks old	Generally 6–8/group for animal experiments; 12 recipients/group for embryo-transfer assessment	Fluorescent PS particles; 0.2, 2, 10, 50, and 100 μm across entry-route experiments; 2 μm in the principal reproductive-toxicity experiments	Route experiments: oral gavage at 10 mg/kg for 30 days, intravenous administration at 40 mg/kg every 2 days for 6 days, or vaginal administration at 10 mg/kg for 14 days. Reproductive experiments: intravenous administration at 20 mg/kg every 2 days for 20 days or oral gavage at 5 mg/kg every 2 days for 3.5 months	Intravenous exposure reduced mean litter size and increased the male-to-female ratio at birth, whereas pup body weight and pregnancy rate were unchanged. After long-term gavage exposure, implantation of transferred normal embryos was reduced, whereas recipient pregnancy rate was not significantly changed	Route- and size-dependent uterine particle entry and deposition; detailed quantitative uterine morphometry was not reported	Long-term gavage exposure increased uterine TNF-α, IL-1β, IL-6, iNOS, and COX-2; uterine entry differed according to particle size and exposure route; embryo-transfer experiments supported a uterine contribution to reduced implantation	Stereofluorescence imaging; H&E; ELISA; Western blotting; embryo-transfer assessment	In mice, uterine entry of fluorescent PS particles varied according to particle size and exposure route. Intravenous exposure reduced litter size and altered offspring sex ratio, whereas long-term gavage exposure induced uterine inflammation and reduced implantation of transferred embryos
5	Sun et al. 2025 [31]	China	C57BL/6 mice (female)	6 weeks old; acclimated for 1 week	6/group	Fluorescent PS-MPs; 5 μm	Oral gavage at 50 mg/kg/day for 14 days; administration continued every 2 days during mating for up to 1 week	Reduced implantation-site number and litter size	Abnormal uterine luminal epithelial proliferation and cell death during the receptive phase; reduced uterine organ coefficient	Reduced uterine receptivity-related gene expression; altered gut–microbiota composition and functional profiles; increased uterine ACSL4 and MDA together with decreased GPX4, SLC7A11, and FTH1, consistent with ferroptosis-related changes	In vivo fluorescence imaging; IF; TUNEL assay; qRT-PCR; Western blotting; metagenomic sequencing; MDA assay	Exposure to PS-MPs impaired uterine receptivity and implantation and was accompanied by gut–microbiota dysbiosis and uterine ferroptosis-related changes, implicating a potential microbiota–ferroptosis link
6	Cary et al. 2025 [32]	USA	Sprague–Dawley rats (pregnant female)	Time-pregnant; exposure from GD5 to GD19	Endpoint-specific; up to 57 controls and 32 exposed dams	Polyamide-12 MNP aerosol; mixed nano- and microscale size distribution characterized in the exposure chamber	Whole-body inhalation at 10.15 ± 1.36 mg/m^3^ for 4 h/day, 5 days/week, from GD5 to GD19	Reduced fetal weight, increased placental weight, and reduced placental efficiency; litter size and resorption number were unchanged	No anatomical differences were detected in uterine radial arteries; uterine histopathology was not assessed	Uterine conduit-artery reactivity was unchanged, whereas endothelium-dependent dilation of uterine radial arteries was impaired; p-eNOS Ser1176 and thioredoxin were reduced and 3-nitrotyrosine was increased; BH4 and DTT partially restored radial-artery dilation ex vivo	Wire myography; pressure myography; Western blotting; oxymyoglobin oxidation assay	Repeated gestational inhalation of polyamide MNPs impaired uterine radial-artery endothelial function through disturbances in eNOS/BH4 and redox signaling and was accompanied by reduced fetal growth and placental efficiency
7	Fournier et al. 2020 [33]	USA	Sprague–Dawley rats (pregnant female)	Time-pregnant; single exposure on GD19	14 saline controls and 11 exposed dams for litter outcomes; a separate cohort of 14 naïve pregnant rats was used for placental perfusion	PS-NPs; nominally 20 nm, with a mean agglomerate diameter of approximately 21.9 nm	Single intratracheal instillation of 2.64 × 10^14^ particles on GD19	Reduced fetal and placental weights and increased resorption sites; litter size was not significantly altered	No placental histopathological alterations were identified after ex vivo perfusion; uterine morphology was not assessed	Translocation from the maternal lung to maternal and fetal tissues, including the placenta and fetal liver, lung, kidney, heart, and brain; ex vivo placental perfusion demonstrated passage into fetal effluent without altered umbilical fluid flow	Fluorescence imaging; enhanced hyperspectral microscopy; ex vivo placental perfusion; H&E	Maternal pulmonary exposure to PS-NPs resulted in placental and fetal particle deposition and reduced fetal and placental weights in late-pregnant rats
8	Hu et al. 2021 [34]	China	BALB/c female mice mated with C57BL/6 males	8–10 weeks old; early pregnancy	9/group	PS-MPs; approximately 10 μm	Intraperitoneal injection of 250 μg in 200 μL saline on GD5.5 and GD7.5	Increased embryo-resorption number and rate; viable embryo number was unchanged	Reduced number and diameter of uterine arterioles at the maternal–fetal interface	Reduced decidual NK-cell proportion; increased placental CD4^+^ T-cell proportion; macrophage polarization shifted toward M2 predominance; altered placental pro- and anti-inflammatory cytokine expression	SEM and FTIR for particle characterization; flow cytometry; H&E; RT-qPCR	Peri-implantation exposure to PS-MPs increased embryo resorption and was accompanied by altered maternal–fetal immune-cell and cytokine profiles and fewer, smaller uterine arterioles, suggesting immune and vascular involvement
9	Li et al. 2025 [35]	China	Kunming mice (female)	8 weeks old; exposure occurred before mating	6/group in control, PS-NP, JNK-inhibitor, and ERK-inhibitor groups	PS-NPs; 50–90 nm	Oral gavage at 1 mg/kg/day for 90 days before mating, with no further gavage after pregnancy was established; JNK or ERK inhibitors were administered intraperitoneally from GD2 to GD7	Reduced implantation-site number; JNK inhibition partially restored implantation, whereas ERK inhibition did not significantly restore implantation	Fusion and edema at implantation sites; reduced formation of polyploid decidual cells	Reduced decidualization and stromal-proliferation indices, including altered BMP2, MMP9, PCNA, Ki67, FOXO1, and Cyclin–CDK proteins; disrupted uterine redox indices; JNK inhibition improved implantation, decidualization, and stromal proliferation, whereas ERK inhibition improved selected redox indices but not implantation	H&E; IHC; IF; Western blotting; qRT-PCR; ELISA	Preconception exposure to PS-NPs impaired implantation and decidualization. JNK inhibition partially rescued implantation, decidualization, and stromal proliferation, whereas ERK inhibition did not restore implantation
10	Amran et al. 2023 [36]	Malaysia	Sprague–Dawley rats (female)	28 days old; prepubertal	32 total; 8/group	PS-MPs; particle size not reported	Oral gavage of PS-MPs at 2.5 mg/kg/day for 6 weeks; the co-treatment group received Kelulut honey at 1200 mg/kg/day 30 min before PS-MP administration	Not measured	Reduced uterine wet and relative weights; reduced height of the luminal and glandular epithelia, endometrial and myometrial thickness, and gland diameter; stromal edema and disorganized myometrial fibers; these changes were attenuated by Kelulut honey co-treatment	Reduced serum E2, progesterone, FSH, and LH; increased uterine ERα and ERβ mRNA and protein expression; Kelulut honey co-treatment attenuated several hormonal, receptor-expression, and histological changes	H&E and histomorphometry; ELISA; qRT-PCR; IHC	Exposure to PS-MPs altered uterine histology, reproductive hormone levels, and ERα/ERβ expression in prepubertal rats. Kelulut honey co-treatment attenuated these alterations, although the protective mechanism was not directly established
11	Wang et al. 2025 [37]	China	Sprague–Dawley rats (nonpregnant and pregnant females)	7 weeks old	Nonpregnant experiment: 8/group; pregnant experiment: 16/group initially, with endpoint-specific sample sizes generally of 5–7/group	Fluorescent PS-NPs; 80 nm	Nonpregnant rats received oral gavage at 0.6, 6, or 60 mg/kg/day for 3 days. Pregnant rats received oral gavage or intraperitoneal injection at the same doses on GD1–3	Implantation-site numbers were unchanged on GD8 and GD13 after oral exposure and on GD8 after intraperitoneal exposure	Dose-related reduction in endometrial glandular epithelial thickness and gland number; reduced primordial follicles together with increased primary, secondary, antral, and atretic follicle proportions	Serum E2 and FSH were reduced; LH increased with dose; progesterone increased only at the low dose and returned toward control levels at medium and high doses; selected ovarian and uterine receptor transcripts were altered; LIF, WNT4, and STAT3 expression was unchanged during pregnancy	H&E; uterine and ovarian morphometry; ELISA; qRT-PCR; fluorescence imaging	Short-term exposure to PS-NPs altered ovarian follicle distributions, uterine histology, and selected hormone and receptor measures in nonpregnant rats but did not alter implantation-site numbers after exposure on GD1–3.

**Table 3:** The contextual details of included in vitro studies.

ID	Study	Location	In Vitro Model Type	Cell/Tissue Characteristics	Replicate Number	Particle Type; Size	Exposure Type	Exposure Mode	Cellular/Reproductive Functional Outcomes	Detection Techniques	Conclusion
1	Arcuri et al. 2025 [18]	Italy	Scaffold-based 3D human endometrial co-culture model	Commercial human endometrial stromal cells (hESCs; passages 7–11) and human endometrial epithelial cells (hEECs; passages 10–14) were co-cultured for 35 days on porous scaffolds	Exposures performed at least in triplicate in three independent experiments	Fluorescent amine-modified PS-MPs; particle size not reported in the article	Acute in vitro exposure	PS-MPs at 0.25, 0.5, 0.75, 1, 10, 12.5, 25, or 50 mg/mL for 24 or 48 h	PS-MPs crossed the epithelial layer and were detected in the stromal compartment from 24 h onward. No significant barrier, viability, or collagen changes occurred after 24 h or after 48 h at 0.25–1 mg/mL. Exposure to 10–50 mg/mL for 48 h reduced cell viability and TEER, disrupted epithelial organization, decreased ZO1 and CDH1 transcription, increased collagen deposition and collagen-gene expression, and altered pro-fibrotic signaling	MTT assay; H&E and Picrosirius red staining; stereological collagen analysis; TEER; confocal microscopy; ZO-1 immunofluorescence; qRT-PCR	Under the tested conditions, high-concentration 48-h PS-MP exposure disrupted epithelial-barrier integrity and induced pro-fibrotic remodeling and collagen deposition in a 3D human endometrial model, whereas shorter or lower-concentration exposures produced no comparable effects.
2	Kim et al. 2025 [38]	Korea	Primary human endometrial stromal cells	Primary eutopic ESCs isolated from endometrial tissues of 13 women aged 25–52 years undergoing surgery for benign gynecological conditions without endometriosis; passages 3–6	At least three independent replicates; endpoint-specific *n* = 3–7	PS-NPs, 100 nm; PS-MPs, 1 and 5 μm; fluorescent 100-nm and 1-μm particles were used for imaging	Acute in vitro exposure	PS particles at 1–10,000 μg/mL for 24 h; 100 μg/mL was used for live-cell imaging and mechanistic assays, and 1 mg/mL for the principal viability comparison	Cellular uptake and cytoplasmic and nuclear accumulation were greater for 100-nm and 1-μm particles than for 5-μm particles. Exposure to 100-nm PS-NPs or 1-μm PS-MPs at 1 mg/mL reduced cell viability, whereas 5-μm PS-MPs did not. At 100 μg/mL, only 100-nm PS-NPs significantly reduced the p-AKT/AKT and p-ERK/ERK ratios and significantly decreased mitochondrial membrane potential; the corresponding effects of 1-μm PS-MPs were not statistically significant	CCK-8; IncuCyte live-cell imaging; confocal microscopy and Z-stack analysis; NucSpot nuclear staining; Western blotting; JC-1 staining	PS particles produced size- and concentration-dependent uptake and cytotoxicity in human ESCs. The 100-nm PS-NPs produced the clearest antiproliferative signaling and mitochondrial-depolarization effects, whereas 5-μm PS-MPs showed no significant toxicity under the tested conditions.
3	Wu et al. 2022 [28]	China	Mouse endometrial epithelial cell line	MEE mouse endometrial epithelial cells cultured in DMEM/F12 containing 10% FBS	At least three independent samples; CCK-8 assay *n* = 6	PS-MPs; 5–10 μm	Acute mechanistic in vitro exposure	MEE cells were co-exposed for 24 h to 500 mg/L PS-MPs with or without the TLR4 inhibitor TLR4-IN-C34 (40 μM), NOX2 inhibitor GSK2795039 (25 μM), or Notch activator VPA (4 mM)	PS-MPs reduced cell viability to approximately 80%, increased ROS and 8-OHdG, activated TLR4/NOX2, Notch, and TGF-β/Smad signaling, and increased collagen and pro-fibrotic markers. TLR4 or NOX2 inhibition reduced oxidative stress, downstream pathway activation, and collagen-related changes. Notch activation with VPA restored downstream Notch/TGF-β and pro-fibrotic responses without restoring ROS production	CCK-8; DCFH-DA ROS assay; immunofluorescence; qRT-PCR; Western blotting; 8-OHdG assay; ADAM and γ-secretase concentration/activity assays	In MEE cells, pharmacological intervention supported the involvement of a TLR4/NOX2–oxidative stress–Notch/TGF-β signaling sequence in PS-MP-induced pro-fibrotic cellular changes
4	He et al. 2025 [16]	China	Primary human endometrial stromal cells	Human ESCs isolated from endometrial tissue; donor number and passage range were not clearly reported for the in vitro experiments	Each group assayed in triplicate	PS-NPs; 50 nm, described as PS microspheres in the source article	Short-term in vitro exposure	Proliferation assay: 0 or 0.5 μg/mL PS-NPs for 7 days. Wound-healing and transwell assays: 0, 0.05, or 0.5 μg/mL for 12–24 h. For pathway assessment, cells were exposed to 0.5 μg/mL PS-NPs, with LY294002 (20 μM) administered 30 min before migration assays	PS-NPs increased ESC proliferation after several days of culture and enhanced wound closure and transwell migration. PS exposure increased AKT phosphorylation, while LY294002 inhibited PS-induced migration, supporting AKT involvement in the migratory response	CCK-8; wound-healing assay; transwell migration assay; Western blotting	Low-concentration 50-nm PS-NP exposure increased proliferation and migration of human ESCs under the tested conditions. Pharmacological inhibition supported AKT involvement in the migration response, but the experiments did not establish that PS-NPs cause endometrial-polyps in vivo
5	Poinsignon et al. 2025 [39]	France	Primary human villous cytotrophoblasts and chorionic-villus explants	Primary VCTs and chorionic villi obtained from term singleton placentas delivered by cesarean section at 37–39 gestational weeks; cultured VCTs spontaneously fused into syncytiotrophoblasts	Endpoint-specific; generally 4–7 independent placentas, with Western blotting *n* = 7 and qRT-PCR *n* = 6	Fluorescent PS-NPs; nominal diameters of 20 and 100 nm	Acute in vitro and ex vivo exposure	Primary VCTs were exposed to 0.01, 1, 10, or 100 μg/mL PS-NPs for 4–48 h; chorionic-villus explants were exposed to 100 μg/mL for 2–24 h	PS-NPs entered trophoblastic cells in a time- and size-dependent manner, with faster translocation of 20-nm particles. PS-NP20 produced earlier ROS generation and greater cytotoxicity, whereas PS-NP100 induced prominent lysosomal and autophagy-related alterations. Pro-inflammatory responses differed by particle size and concentration. hCG secretion decreased after 0.01 μg/mL PS-NP20 and dose-dependently after 1–100 μg/mL PS-NP100. VCT-to-ST fusion and PlGF secretion were not significantly altered	WST-1; CM-H2DCFDA; confocal microscopy; TEM; immunofluorescence; qRT-PCR; Western blotting; lysosomal and autophagy assays; hCG and PlGF assays; fusion-index assessment	Acute PS-NP exposure induced size- and concentration-dependent internalization, cytotoxic, oxidative, inflammatory, lysosomal, and endocrine responses in primary human trophoblast models. These cellular findings do not by themselves establish adverse pregnancy outcomes.
6	Qin et al. 2024 [17]	China	Human endometrial organoids	Human endometrial epithelial gland cells isolated from endometrial tissue and cultured as Matrigel-embedded organoids	At least three experimental repeats; the number of tissue donors used to generate organoids was not clearly reported	Fluorescent PS-MPs; 2 μm	Acute organoid exposure	Human endometrial organoids were exposed to 5 or 50 μg/mL PS-MPs for 48 h	Exposure to 50 μg/mL PS-MPs caused distorted organoid morphology, collapse of cellular boundaries, and markedly increased Annexin-V fluorescence and apoptosis. Comparable toxicity was not clearly demonstrated at 5 μg/mL. Endometrial-receptivity markers and implantation function were not assessed	Bright-field microscopy; fluorescence microscopy; Annexin-V-FITC staining and apoptosis-index quantification	High-concentration 2-μm PS-MP exposure disrupted growth morphology and induced apoptosis in human endometrial organoids under the tested conditions; the experiment did not directly assess endometrial receptivity or implantation.

## Data Availability

The raw data supporting the conclusions of this article will be made available by the authors on request.

## Supplementary Materials

The following supporting information can be downloaded at: https://www.mdpi.com/article/10.3390/toxics14080677/s1, Table S1: Detailed search strategies; Table S2: TRAEC scoring results from Assessor 1; Table S3: TRAEC scoring results from Assessor 2.

## Abbreviations

The following abbreviations are used in this manuscript:

MNPs	micro- and nanoplastics
TRAEC	Targeted Risk Assessment of Environmental Chemicals
PS-NPs	polystyrene nanoplastics
ROS	reactive oxygen species
MPs	microplastics
NPs	nanoplastics
IVF-ET	in vitro fertilization-embryo transfer
LDIR	laser direct infrared spectroscopy
Py-GC/MS	pyrolysis–gas chromatography/mass spectrometry
PA	polyamide
PU	polyurethane
PET	polyethylene terephthalate
PS-MPs	polystyrene microplastics
hESCs	human primary endometrial stromal cells
CES	Comprehensive Evidence Score
PE	polyethylene
PS	polystyrene
PVC	polyvinyl chloride
PP	polypropylene
uNK cells	uterine natural killer cells
