# Supplementary material for: Effects of Exposure to Micro- and Nanoplastics on Endometrial Injury and Adverse Pregnancy Outcomes: Evidence Integration Using the Targeted Risk Assessment of Environmental Chemicals Framework
Source: Toxics. 2026 Jul 31;14(8):677. doi: 10.3390/toxics14080677 (PMC13517212; doi:10.3390/toxics14080677)
Supplement: Supplementary file 1 [file toxics-14-00677-s001.zip › Supplementary Table S1.pdf]

Supplemental Table S1: Detailed Search Strategy

PubMed

Search strategy:

#1 "Endometrium"[Mesh] OR "Uterus"[Mesh] OR "Embryo Implantation"[Mesh] OR endometrial receptiv*[tiab] OR endometrium[tiab] OR endometria[tiab] OR uterus[tiab] OR uteri[tiab] OR uterine[tiab] OR womb[tiab] OR wombs[tiab] OR "uterine fundus"[tiab] OR "fundus uteri"[tiab] OR "uterine cornua"[tiab] OR "embryo implantation"[tiab] OR "blastocyst implantation"[tiab] OR "ovum implantation"[tiab] OR nidation[tiab] OR decidual*[tiab] OR "decidual cell reaction*"[tiab]

#2 "Microplastics"[Mesh] OR nanoplastic*[tiab] OR "nano-plastic*"[tiab] OR "nano plastic*"[tiab] OR "nano-ps"[tiab] OR microplastic*[tiab] OR "micro-plastic*"[tiab] OR "micro plastic*"[tiab] OR "polystyrene nanoplastic*"[tiab] OR "polystyrene microplastic*"[tiab] OR "plastic nanoparticle*"[tiab] OR "plastic nanoparticles"[tiab] OR "polystyrene microbead*"[tiab] OR "plastic microbead*"[tiab] OR "plastic microparticle*"[tiab] OR "polyethylene microplastic*"[tiab] OR "polypropylene microplastic*"[tiab]

#3 #1 AND #2

Numbers: 113

Web of Science

Search strategy:

TS=(
(
"endometrial receptiv*" OR endometrium OR endometria
OR uterus OR uteri OR uterine OR womb OR wombs
OR "uterine fundus" OR "fundus uteri" OR "uterine cornua"
OR "embryo implantation" OR "blastocyst implantation" OR "ovum implantation"
OR nidation OR decidual* OR "decidual cell reaction*"
)
AND
(
nanoplastic* OR "nano-plastic*" OR "nano plastic*" OR "nano-ps"
OR microplastic* OR "micro-plastic*" OR "micro plastic*"
OR "polystyrene nanoplastic*" OR "polystyrene microplastic*"
OR "plastic nanoparticle*" OR "plastic nanoparticles"
OR "plastic microbead*" OR "polystyrene microbead*"
OR "plastic microparticle*"
OR "polyethylene microplastic*" OR "polypropylene microplastic*"
)
)

Embase

Search strategy:

(
'endometrium'/exp OR 'uterus'/exp OR 'embryo implantation'/exp
OR endometrial receptiv*:ti,ab,kw
OR endometrium:ti,ab,kw OR endometria:ti,ab,kw
OR uterus:ti,ab,kw OR uteri:ti,ab,kw OR uterine:ti,ab,kw OR womb:ti,ab,kw OR wombs:ti,ab,kw
OR 'uterine fundus':ti,ab,kw OR 'fundus uteri':ti,ab,kw OR 'uterine cornua':ti,ab,kw
OR 'embryo implantation':ti,ab,kw OR 'blastocyst implantation':ti,ab,kw OR 'ovum implantation':ti,ab,kw
OR nidation:ti,ab,kw OR decidual*:ti,ab,kw OR 'decidual cell reaction*':ti,ab,kw
)
AND
(
'microplastic'/exp OR 'nanoplastic'/exp
OR nanoplastic*:ti,ab,kw OR "nano-plastic*":ti,ab,kw OR "nano plastic*":ti,ab,kw OR "nano-ps":ti,ab,kw
OR microplastic*:ti,ab,kw OR "micro-plastic*":ti,ab,kw OR "micro plastic*":ti,ab,kw
OR "polystyrene nanoplastic*":ti,ab,kw OR "polystyrene microplastic*":ti,ab,kw
OR "plastic nanoparticle*":ti,ab,kw OR "plastic microbead*":ti,ab,kw OR "polystyrene microbead*":ti,ab,kw
OR "plastic microparticle*":ti,ab,kw
OR "polyethylene microplastic*":ti,ab,kw OR "polypropylene microplastic*":ti,ab,kw
)

Numbers: 141

Scopus

Search strategy:

TITLE-ABS-KEY(
(
"endometrial receptiv*" OR endometrium OR endometria
OR uterus OR uteri OR uterine OR womb OR wombs
OR "uterine fundus" OR "fundus uteri" OR "uterine cornua"
OR "embryo implantation" OR "blastocyst implantation" OR "ovum implantation"
OR nidation OR decidual* OR "decidual cell reaction*"
)
AND
(
nanoplastic* OR "nano-plastic*" OR "nano plastic*" OR "nano-ps"
OR microplastic* OR "micro-plastic*" OR "micro plastic*"
OR "polystyrene nanoplastic*" OR "polystyrene microplastic*"
OR "plastic nanoparticle*" OR "plastic nanoparticles"
OR "plastic microbead*" OR "polystyrene microbead*"
OR "plastic microparticle*"
OR "polyethylene microplastic*" OR "polypropylene microplastic*"
)
)

Numbers: 102

Summary

Database	Results
PubMed	113
Web of Science	126
Embase	141
Scopus	102
